# From endorsement of the WHO Acceleration Plan to Stop Obesity to national implementation: country progress on health system preparedness to scale up a comprehensive obesity chronic care programme

**DOI:** 10.1016/S2214-109X(25)00496-6

**Published:** 2026-03-03

**Authors:** Francesca Celletti, Claire Chaumont, Jørgen Torgerstuen Johnsen, Francesco Branca, Anna Wright, Jennifer Manne-Goehler, Melanie Bertram, Rupasree Srikumar, Pavel Ursu, Luz Maria De-Regil

**Affiliations:** a Department of Nutrition and Food Safety, WHO, Geneva, Switzerland; b Harvard T H Chan School of Public Health, Boston, MA, USA; c Institute of Global Health, University of Geneva, Geneva, Switzerland; d The London Borough of Camden, Camden Town Hall, London, UK; e Division of Infectious Diseases, Brigham and Women's Hospital, Harvard Medical School, Boston, MA, USA; f Department of Data, Digital and Artificial Intelligence, WHO, Geneva, Switzerland; g WHO Nigeria Country Office, Abuja, Nigeria

## Abstract

Addressing the global obesity crisis requires health systems that move beyond prevention to include care and treatment. However, translating global policy into national implementation remains challenging. Through the WHO Acceleration Plan to Stop Obesity, 34 countries committed to reducing the prevalence of obesity by 5% by 2030. Using the plan's operational model, we applied a policy and impact cycle and created a 100-day challenge platform, to support 12 countries to integrate and scale chronic obesity care within their health systems. This paper captures the approaches, system design, progress, and lessons in expanding access to chronic obesity care across the life course. Results show that political commitment, structured implementation, and targeted technical support enabled rapid progress in service design and delivery readiness. Stakeholder engagement, community participation, and data-driven planning emerged as key enablers of success. The countries in this study provide a blueprint for embedding obesity care at scale, underscoring the need for a coordinated global response.

## Introduction

### A lesson from recent history

Obesity is affecting wellbeing and lives at an ever-increasing rate all over the globe. Classified by WHO as a chronic disease, obesity has many negative impacts on health, often contributing as a risk factor for other diseases.^1^ In 2021, there were an estimated 3·7 million obesity-related deaths from non-communicable diseases, such as cardiovascular diseases, diabetes, cancers, neurological disorders, chronic respiratory diseases, and digestive disorders.^2^ In the last 20–30 years, efforts to respond to the obesity pandemic have focused almost exclusively on public health policies that embed healthy diets (through regulating the availability and marketing of unhealthy foods, implementing fiscal policies, such as sugar-sweetened beverage taxation, front-of-pack labelling, and norms and standards for foods available in schools) and regular physical activity as the most accessible, available, and affordable behaviours of daily life. Although there is good evidence on the effectiveness of a range of such policies in preventing obesity and other chronic diseases across several contexts,^3^, ^4^ multisectoral efforts to influence behaviours around healthy diets and physical activity have, so far, been insufficient to substantially reduce obesity-related morbidity and mortality.^5^

Drawing on lessons from other large-scale health programmes, real progress in scaling any chronic care programme comes from integrated, system-wide approaches. In Mexico, embedding diabetes care into primary health services improved glucose control beyond earlier, lifestyle-only strategies.^6^, ^7^ Brazil's Family Health Strategy, supported by community-based teams, led to better management of hypertension and diabetes and reduced hospitalisations.^8^, ^9^ Egypt's national hepatitis C campaign screened over 50 million people and treated millions with affordable antivirals, cutting prevalence from 15% in 2015 to under 1% in 2023.^10^ These examples show that success requires a combination of prevention, coordinated care, accessible treatment, and strong primary health systems. A similar health system preparedness response, aligned with preventive public health policies, must be viewed as an essential component of a comprehensive approach that can have a meaningful impact on the obesity pandemic—a component that has been missing to date.

### Strengthening the health system obesity response

Beyond having a direct effect on patient care, a health system that is prepared to respond to obesity through screening, early diagnosis, care, support, and treatment services will also promote action, reduce stigma, and unlock resources across other sectors, as was the case in the response to HIV/AIDS, COVID-19, viral hepatitis, sexually transmitted infections, and tuberculosis.

Leveraging health systems is key to accelerating the response to obesity. Health systems are uniquely positioned to engage large populations on prevention and care and reduce weight-related stigma. They can also create appropriate demand for treatment and systemic change, advocating for fairer access to effective therapies, such as GLP-1 receptor agonists,^11^ through mechanisms such as market shaping and innovative financing, and learning from HIV and tuberculosis responses. When aligned with broader actions in food policy, urban planning, and poverty reduction, health systems can generate powerful, multisector synergies that foster healthier environments.

### Opportunities for a stronger response

Until recently, obesity care has been fragmented, hard to scale, and often deprioritised due to few treatment options, workforce constraints, and persistent stigma. But novel therapies such as GLP-1 receptor agonists are changing this landscape, increasing demand for pharmacological interventions for weight loss, and sparking political and commercial attention. This momentum presents a substantial opportunity to expand access and embed obesity services within primary care. Although a purely medicalised, tertiary-care level model risks being inequitable and unsustainable, a comprehensive health system response that makes prevention, care, and treatment universally available, affordable, and integrated into routine care is what is needed.

At the 75th World Health Assembly in 2022, member states adopted the WHO Acceleration Plan to Stop Obesity, a systematic framework for countries to implement obesity prevention and management policies.^12^ Centred around the forthcoming WHO Adopt, Create, and Transform (ACT) technical package (panel 1), the plan provides evidence-based interventions and tools to support implementation across food systems, physical activity, and health services.

Panel 1 **The WHO Adopt, Create, and Transform (ACT) technical package**

**Figure 1:**
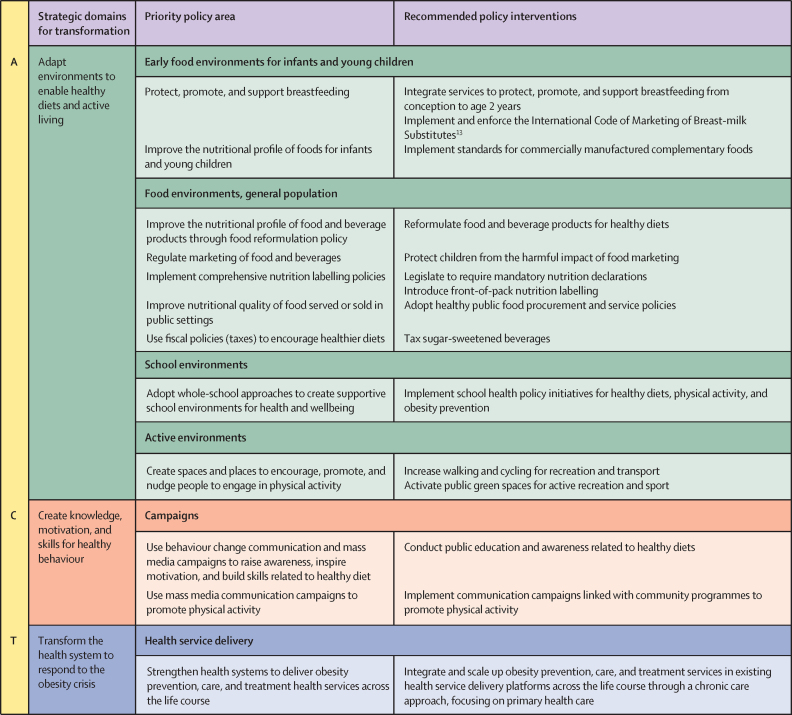
The WHO Adopt, Create, and Transform (ACT) framework

The forthcoming WHO ACT technical package to stop obesity has been developed through a consultative process, distilling the technical expertise at all levels of WHO across a wide range of health topics and consolidating the knowledge and experience of pioneer countries through pilot testing. It is intended to support national policy makers, programme managers, and implementers to deliver concrete action. It focuses on proven interventions to be selected by countries according to their own priorities and tailored to the local context. It aims to help provide detail on how to prepare, develop, and implement recommended obesity prevention and management policies, and monitor and evaluate these policies to ensure the use of data and concrete progress on the ground. At the core of the technical package is the ACT framework comprised of three broad strategic areas for transformation with 15 recommended interventions (figure 1).

The current challenge is translating global commitments into national action, particularly through scaling up health system responses that provide access to diagnostics, effective medicines, and surgery, where appropriate. 34 countries have committed to the plan, aiming to reduce national obesity prevalence by 5% by 2030. Of these countries, 12 have prioritised building their health systems' response. WHO is supporting these 12 countries to expand prevention, care, and treatment by use of a primary health-care and life course approach. This includes rolling out multimodal chronic care models with structured lifestyle interventions, access to emerging pharmacological treatments, and surgical options when needed. This Health Policy outlines the policy implementation approach in these 12 countries, detailing the health system design, operational methods, progress to date, and early lessons learned.

## Methods

### The WHO Acceleration Plan to Stop Obesity operational model

**Figure 2:**
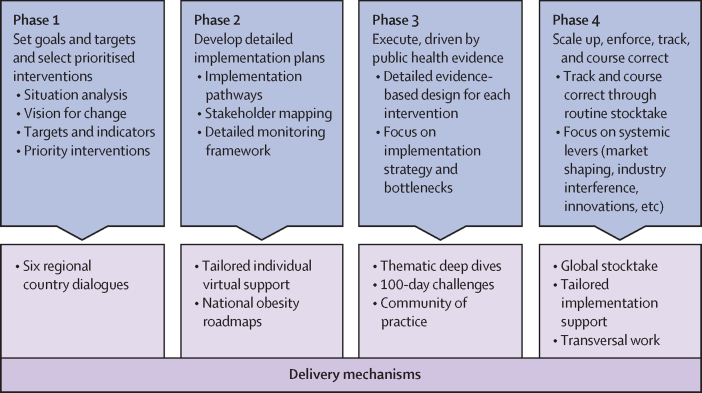
The WHO Acceleration Plan to Stop Obesity operational model

The acceleration plan focuses on one ambitious goal: to stop the rising obesity pandemic. Endorsed at the 75th World Health Assembly in 2022, the plan is grounded in three key pillars: shaping the political environment, creating a platform for delivery, and strengthening accountability.^12^ All 34 front-runner countries endorsing the plan are following the same operational method, which provides a systematic process to support progress and achieve impact across different interventions.^14^ This model is characterised by policy and impact cycles that blend policy making with implementation and delivery science to track progress, make course corrections, and redesign when needed to ensure targets are achieved. It uses simplified indicators and employs pragmatic accountability rubrics for national and global reporting. Implementation support for country teams; country progress reports; global stocktake to review progress across all countries; and advocacy and mobilisation to engage more stakeholders, communities at large, and people living with obesity are the metrics envisaged to inform the accountability report to the World Health Assembly every 2 years. The operational model for the acceleration plan, which is based on the WHO Implementation Playbook,^15^ is summarised in figure 2 and will be described in further detail in the forthcoming WHO and UNICEF operational model document.

### Implementation

Phases 1 and 2 of the plan (figure 2) were implemented across all 34 front-runner countries. These initial phases aimed to develop a shared, ambitious vision for addressing obesity by 2030, articulate a practical pathway forward, and build the political and advocacy case needed to generate national momentum. Activities during this phase included high-level political engagement, situation analyses, and initial policy prioritisation guided by the WHO technical package (panel 1).

Phase 3 focused on intervention-specific implementation. 12 countries (Botswana, Brunei, Egypt, Ireland, Jordan, Malaysia, the Philippines, Portugal, Slovenia, South Africa, Spain, and Türkiye) opted to prioritise the integration and scale-up of chronic care for obesity within their health systems. These countries received intensified technical support to strengthen government structures and health system capacity for sustained delivery of obesity chronic care through the life course and following a primary health-care approach. The process included an initial in-depth, deep dive workshop tailored to each country's context, followed by participation in a structured community of practice to move to execution through a 100-day challenge. The challenge provided ongoing technical assistance, peer learning, and capacity-building from November, 2022, to December, 2024.

In Phase 3, ten of the 12 countries that had selected the health systems preparedness priority intervention participated in an inter-regional WHO-led workshop and 100-day challenge. The two remaining countries, Egypt and Spain, instead received bilateral 1:1 support. The workshop enabled an in-depth immersion into the features of national health system integration and scale-up of obesity prevention, care, and treatment services. The 100-day challenge included five virtual sessions, during which participating countries engaged and worked towards four defined milestones: map services through the WHO Universal Health Coverage Service Planning Delivery and Implementation Platform,^16^ create a first draft of gap analysis in the scope of practice, present a draft plan and budget, and develop process and outcome indicators. Countries used this opportunity to refine national plans, address early implementation barriers, and validate service delivery models. Coaching and peer-learning sessions helped countries adapt the intervention to context-specific needs and address challenges identified during the virtual sessions. In the country engagement, emphasis was placed on the importance of engagement with obesity-related community organisations and people living with obesity. Involving people living with obesity and the community was intended as a collaborative process to foster the participation and empowerment of communities in the design, implementation, and evaluation of health policies and programmes to improve health outcomes.^17^ Panel 2 outlines the steps taken during the workshop and further used during the 100-day challenge sessions. From this point forward, countries were proceeding to nationwide implementation, systems transformation, and accountability according to their 2025–30 national obesity roadmap. Further results will be reported in the future.

Panel 2 **Phase 3 steps undertaken in the WHO workshop and 100-day challenge sessions**

**Policy preparation to lay the foundation for the coordinated integration of a national obesity chronic care programme within the broader health system response**

• Establish governance structures: multisectoral national teams were formed, anchored within non-communicable diseases and nutrition departments, with the inclusion of primary health care and universal health coverage experts to ensure a health systems perspective
• Conduct situational analysis: country teams assessed their health system's readiness and service availability including obesity prevention, care, and treatment within universal health coverage and primary care benefit packages
• Stakeholder mapping and engagement: teams identified key actors, analysed influence and interest, and designed tailored engagement and political mobilisation strategies
• Develop theory of change and issue analysis: countries articulated a shared vision for obesity care, mapped root causes with issue trees, and defined pathways to achieve national outcomes
• Launch a communications and advocacy strategy: strategic efforts were initiated to raise awareness, combat stigma, and build high-level consensus for obesity as a chronic, treatable condition

**Policy and model development to develop a scalable model for health service delivery**

• Finalise national policy framework: countries refined their theory of change and identified service integration points across levels of care
• Design and adapt the model of care: using the WHO Health Service Delivery Framework for Prevention and Management of Obesity,^18^ teams adapted models to include early intervention, prevention, and long-term management, shifting away from reactive care triggered by comorbidities
• Ensure access and financial protection: teams defined strategies to overcome access barriers and expand obesity services under universal health coverage or insurance schemes
• Strengthen monitoring and data systems: indicators were defined, data systems aligned, and mechanisms for continuous feedback and learning were introduced

**Implementation planning and calibration to establish operational readiness and planning for scale-up**

• Develop the national scale-up plan: countries created roadmaps for service expansion, aligned with subnational systems and capacities
• Health workforce capacity building and systems readiness: teams analysed gaps in human resources, scope of practice, referral mechanisms, and supply chains using the WHO Universal Health Coverage Service Planning Delivery and Implementation Platform.^16^ The platform is designed to support countries in developing well designed national health service packages within the context of a broad universal health coverage reform or for a specific disease or risk factor across all levels of a health system
• Ongoing coordination, engagement, and peer learning: cross-country collaboration and communities of practice were fostered to share real-world experiences, troubleshoot bottlenecks, and build collective momentum

## Reporting

To measure progress in integrating and scaling up chronic obesity care programmes, nine key performance indicators (KPIs) were defined, reflecting crucial domains across the scale-up pathway, from governance and policy development to health system readiness, service delivery expansion, and monitoring and evaluation (panel 3). These indicators were grounded in the WHO Health Service Delivery Framework,^18^ and the WHO Implementation Playbook,^15^ ensuring alignment with global standards for the design and scale-up of chronic care services. They also draw on lessons from large-scale chronic disease programmes, including for hypertension, diabetes, and HIV care.^19^, ^20^

Panel 3 **Key performance indicators**

1 Integration of obesity chronic care programmes into national health sector plans
2 Engagement of communities and people living with obesity
3 Integration of obesity prevention, care, and treatment into primary care benefit packages
4 Availability of national clinical guidelines for obesity management and treatment
5 Development of monitoring and evaluation frameworks, including obesity chronic care registries
6 Training of health-care providers in obesity chronic care
7 Availability of anti-obesity medicines, including GLP-1 receptor agonists
8 Provision of obesity health services free at the point of delivery
9 Scale-up of obesity chronic care programmes

The KPIs were designed to capture the essential system-level changes required to scale obesity services effectively, sustainably, and equitably. They were applied during the 100-day challenge to guide progress reviews, identify system bottlenecks, and inform technical support needs. Countries reviewed and validated the indicators through structured group discussions and electronic feedback to ensure their relevance and feasibility for national monitoring.

During each 100-day challenge virtual session, country teams reported progress on KPIs corresponding to the session's milestone, while the WHO Secretariat provided technical clarifications and conducted internal cross-reviews to ensure consistency and comparability. When countries were unable to join the sessions, bilateral follow-up meetings and email exchanges were conducted to secure their input. To strengthen data reliability, country-reported information was triangulated, in consultation with countries, with evidence from published and grey literature and data drawn from national health information systems and national health sector plans. WHO teams jointly reviewed submissions for completeness, plausibility, and internal consistency, and followed up with national government representatives to verify any discrepancies during the 100-day challenge sessions. At the end of this process, all countries verified the reported results with their stakeholders at the country level. Finally, a consolidated table of country progress was shared with all countries for final feedback and validation. This iterative, participatory approach strengthened shared understanding, enhanced data reliability, and fostered cross-country learning.

## Progress results

**Figure 3:**
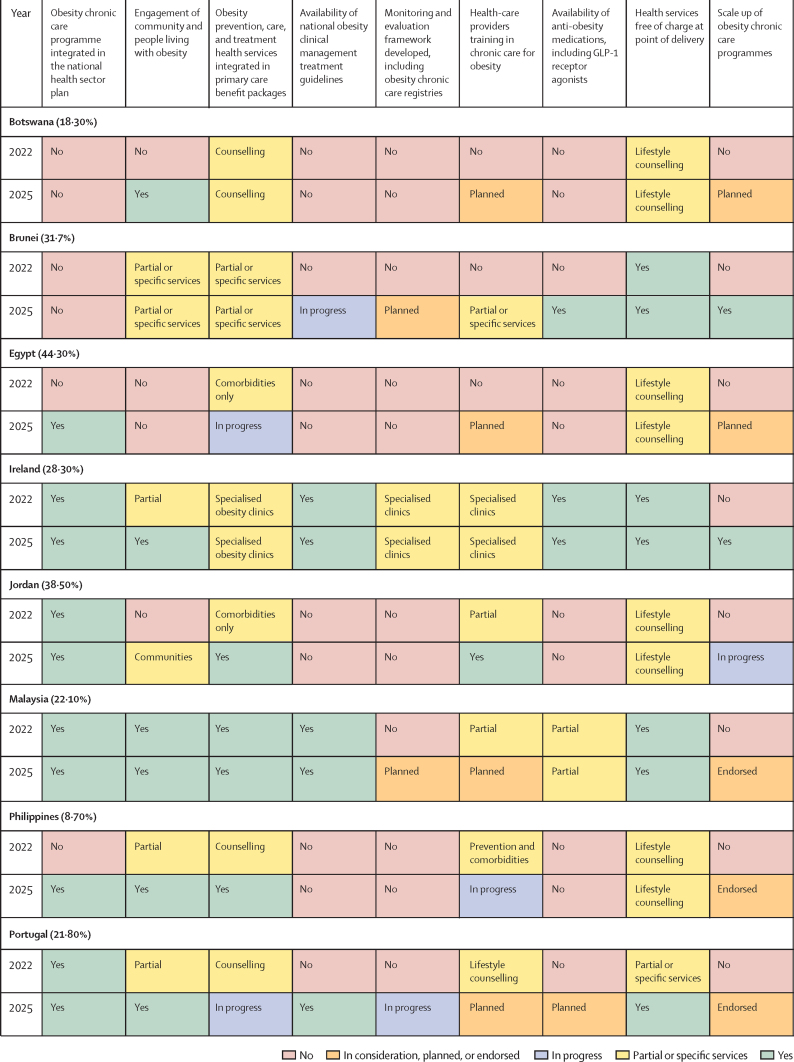

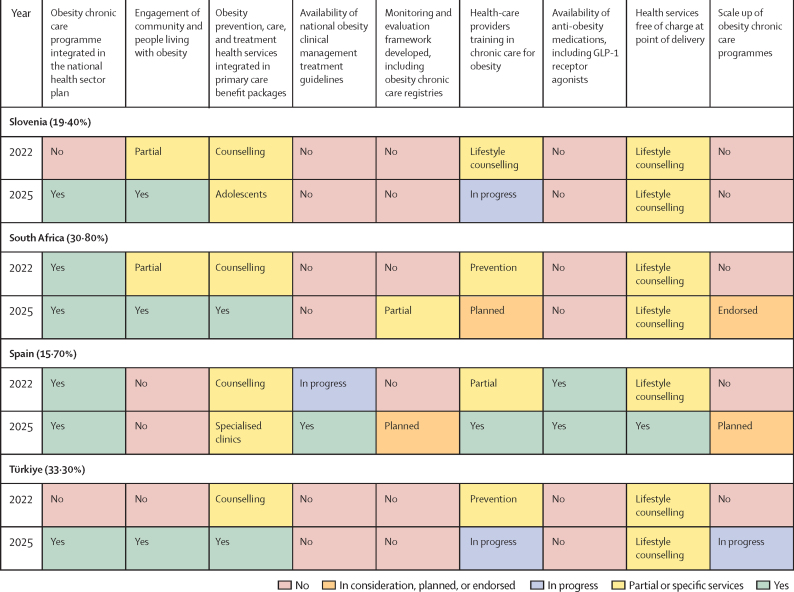
Self-reported country progress against nine key indicators of health system preparedness The prevalence of obesity in adults (age >18 years) in 2022 is provided in parantheses after each country. In consideration, planned, or endorsed (orange) indicates that a policy or service has been formally acknowledged by government or stakeholders and is under active consideration or in the planning or endorsement stage but is not yet operationalised. In progress (blue) reflects that implementation has begun, with activities under way that go beyond planning (eg, workforce training, service roll-out, resource allocation). Partial or specific services (yellow) denote situations where a measure has been implemented in a limited scope (eg, only selected health services).

Figure 3 presents the 2022 baseline position and 2025 status on the nine KPIs. Baseline metrics were self-reported by each of the 12 countries as they embarked on Phase 3 of the acceleration plan. Countries are listed alongside the latest national obesity prevalence in adults (age >18 years) as reported in The State of Food Security and Nutrition in the World 2025.^21^ There has been clear progress against some or all indicators compared with the 2022 baseline, although progress is inconsistent across countries. Progress against each indicator is detailed in the following sections.

### Obesity chronic care programme integrated in the national health sector plan

At baseline, six countries (Ireland, Jordan, Malaysia, Portugal, South Africa, and Spain) had reported on obesity chronic care programmes as part of their national health sector plan. By June, 2025, an additional four countries (Egypt, the Philippines, Slovenia, and Türkiye) integrated obesity chronic care programmes in their national health sector plan. Portugal further strengthened their model of care by legally mandating an integrated model of care for obesity prevention and management, emphasising early identification and treatment at the primary care level. The model includes training for health professionals, awareness raising for primary care physicians, and financial incentives for health-care units to support implementation across the national health system. Botswana reported this KPI as a key missing area. Brunei strengthened political commitment to address obesity by establishing its first obesity task force and integrating management services into primary health care for all age groups. It launched family-oriented initiatives to promote healthy lifestyles and was developing a national obesity response strategy, with health-care provider training planned by January, 2026.

### Engagement of community and people living with obesity

The self-reported progress in this area was positive, with six countries (Botswana, Ireland, Portugal, Slovenia, South Africa, and Türkiye) of 12 reporting action, an increase from a baseline of one (Malaysia). Those countries that are actively engaging reported positively on the value of the stakeholder mapping exercise to increase assessment, outreach, and engagement in the design of the service delivery, or in generating community demand. South Africa has shown strong political commitment to tackling obesity, embedding management into its national strategic plan for non-communicable diseases and primary health-care services. Initial services are delivered through health centres, with provider training via the adult primary care and community health-worker programmes. A strategy for scaling up comprehensive obesity services has been endorsed, and the use of anti-obesity medicines, including GLP-1 receptor agonists, is under review. Through the multisectoral Move for Health programme, the government partners with education, social, and cultural sectors and community running club Parkrun to promote healthier lifestyles. The remaining countries partially engaged with communities and, in the case of Brunei, this engagement was restricted to specific services such as Lifestyle Centres, which offer integrated weight management programmes and early detection of non-communicable disease risk factors.

### Obesity prevention, care, and treatment health services integrated in primary care benefit packages

Four countries (Jordan, the Philippines, South Africa, and Türkiye) made progress in integrating obesity prevention, care, and treatment health services into primary care benefit packages. Egypt and Portugal reported progress in integration, expanding available health services and access to a broader cohort of patients. Slovenia managed integration for adolescents, and Ireland has integrated obesity prevention, care, and treatment within specialised obesity clinics. Spain is committed to tackling childhood obesity through primary care and promoting public health policies aimed at overcoming the social determinants of childhood obesity.

### Availability of national obesity clinical management treatment guidelines

Two countries (Ireland and Malaysia) reported having national clinical guidelines for obesity management and treatment at the 2022 baseline. By 2025, Portugal and Spain had developed clinical guidelines and Brunei was in progress, as of 2025, through its obesity task force.

### Monitoring and evaluation framework developed, including obesity chronic care registries

A monitoring and evaluation framework that includes obesity chronic care registries is an area in which progress has been slow and challenging. Only Portugal reported that they are in progress. Brunei, Malaysia, Portugal, and Spain were planning their monitoring and evaluation frameworks in 2025 and South Africa reported it being partially developed. In Ireland, a framework has been developed for specialised clinics only.

### Health-care providers training in obesity chronic care

By 2025, training was planned or in progress in eight countries: Botswana, Egypt, Malaysia, the Philippines, Portugal, Slovenia, South Africa, and Türkiye. In Ireland, health-care providers receive training in specialist clinics. Jordan and Spain reported going from training of health workers limited to lifestyle counselling to a comprehensive package including prevention, care, and treatment. Brunei had only implemented training programmes for specific services at the tertiary level.

### Availability of anti-obesity medications

At baseline, in 2022, anti-obesity medications, including GLP-1 receptor agonists, for treatment of obesity were available only in Ireland and Spain, on a limited basis. In 2025, the availability of anti-obesity medications to treat obesity as part of national health insurance plans remained in the very early stages, with only Brunei offering anti-obesity medications of any kind free at the point of treatment. Self-reported high costs and poor availability prohibited wider access in the remaining countries. In 2025, Portugal was in the planning phase, as anti-obesity medications were not yet covered or reimbursed under the health-care system for the treatment of obesity. Countries attributed slow progress on this KPI to the still limited availability of comprehensive clinical guidelines for obesity prevention, care, and treatment, which, in 2025, were present in only Ireland, Spain, and Brunei.

### Health services free of charge at point of delivery

Despite the desire for a free at point-of-care model from all 12 countries, most were yet to achieve full financial coverage for a comprehensive set of obesity health services. Of the 12 countries completing Phase 3, five countries (Brunei, Ireland, Malaysia, Portugal, and Spain) provided comprehensive prevention, care, and treatment obesity services free at the point of delivery, with the remaining countries limiting their free provision to lifestyle counselling.

### Scale-up of obesity chronic care programmes

Eight countries (Botswana, Egypt, Jordan, the Philippines, Portugal, South Africa, Spain, and Türkiye) of the 12 reported progress with national scale-up either being planned, endorsed, or in progress, compared with no action in 2022. Brunei and Ireland were the only two countries that reported scale-up plans for their obesity chronic care programmes.

In summary, positive results and progress were observed. Ten countries integrated obesity chronic care into national health plans, with Portugal mandating an integrated model and Brunei establishing an obesity task force. Community engagement, health-care provider training, and integration into primary care benefit packages expanded, and nine countries are advancing national scale-up of obesity programmes. Challenges remain in ensuring broad access to anti-obesity medications, implementing comprehensive monitoring and evaluation frameworks, and providing full financial coverage for obesity prevention, care, and treatment services.

## Discussion

The insufficiency of obesity prevention, care, and treatment is not confined to low-income or middle-income countries; it is a global failure, reflecting systemic neglect of obesity as a chronic disease. The experiences of these front-runner countries in the acceleration plan show that transforming health systems to address obesity is feasible across diverse contexts and income levels. Early results show that, when countries can rapidly secure political commitment, adopt a structured, data-driven methodology, mobilise stakeholders, and build the foundations for integrating obesity services into existing health platforms, progress is possible.

### Progress to date

Countries reported advances in several domains of health-system preparedness. In addition, although high-income countries such as Ireland and Portugal were more advanced at baseline, other countries, such as Brunei, South Africa, and the Philippines managed to secure substantial policy gains during this short timeframe. Political commitment was secured at the highest levels, creating enabling policy environments and engaging civil servants and health system leaders. Stakeholder mapping was conducted across all settings, supporting multisectoral collaboration, and many countries initiated community engagement, including with people living with obesity, although implementation was uneven. Each country developed a scale-up strategy, leveraging existing national health sector plans, universal health coverage roll-outs, and nutrition or obesity programmes to embed services across the life course.

### Persistent challenges

Despite this progress, several gaps in health-system preparedness emerged. First, not recognising obesity as a chronic disease hampers investment and constrains the development of comprehensive models of care. Most countries retain a prevention-focused approach, with few addressing the needs of people with the disease, despite the incremental presence of effective therapies such as GLP-1 therapy. Second, health-care provider capacity is inadequate: few health workers are trained to diagnose, manage, and treat obesity and its comorbidities, and existing training materials are outdated. Third, system-level fragmentation between sectors, programmes, and national versus subnational implementation impedes scale-up. Fourth, financing remains a major barrier. Unlike other major health challenges, such as HIV, tuberculosis, malaria, or vaccine-preventable diseases, no dedicated global financing mechanisms exist for obesity, leaving countries reliant on ad hoc and unsustainable funding streams.

Although WHO classifies obesity as a chronic disease,^1^ and the current body of evidence clearly supports this position,^22^, ^23^, ^24^ some countries continue to view obesity primarily as a risk factor for other conditions rather than a standalone disease. Perceptions and attitudes towards obesity therefore remain divergent. Compared with other non-communicable diseases, knowledge gaps remain in fully understanding the biological and genetic determinants of obesity,^25^ and recognising its impact on the broader burden of non-communicable diseases.^26^

### Addressing the gaps

Several priorities are clear. Embedding recognition of obesity as a chronic disease into national policy is essential to unlock investment and shift from weight loss-focused interventions to lifelong, person-centred care. Accelerated development of national clinical guidelines, informed by forthcoming WHO recommendations on childhood and adolescent obesity management and the use of GLP-1 therapy in adults, will help standardise care.^27^ However, challenges related to access, availability, affordability, sustainability, and equitable access to obesity medicines are expected to persist across countries of all income levels. Expanding registries with trackable clinical indicators would strengthen accountability and support data-driven delivery.

Training programmes for health professionals must be expanded and updated to incorporate competencies in chronic obesity care. Our findings indicate that lifestyle counselling is the most frequently available service. Although counselling is a valuable entry point, its effectiveness depends on delivery within a comprehensive, long-term programme that is responsive to individual goals and motivations. Current WHO guidance^27^ indicates that counselling should not be applied in isolation but rather as the foundation of intensive behavioural therapy within a structured, stepwise approach to obesity management.

Experience from other large-scale health responses provides a roadmap. Political commitment and global level leadership and advocacy, as seen in HIV (through The US President's Emergency Plan for AIDS Relief and The Global Fund to Fight AIDS, Tuberculosis and Malaria), hepatitis C (scale-up of direct-acting antivirals), and immunisation (via Gavi, the Vaccine Alliance), are essential to create enabling environments and mobilise resources.^28^, ^29^, ^30^ Early and inclusive stakeholder mapping and engagement helps build consensus across ministries, professional bodies, and sectors, avoiding the fragmentation that has slowed progress in obesity care. Community mobilisation is another proven driver of success; as shown in HIV and immunisation programmes, empowering affected communities to advocate for their rights, co-design services, and hold systems accountable generates demand and reduces stigma.^31^ Structured, data-driven delivery methods, including registries and measurable indicators, enable real-time tracking and adaptive management—an approach used effectively in chronic care programmes for hypertension and in large-scale immunisation campaigns. Dedicated financing mechanisms are indispensable for scaling prevention, care, and access to medicines as shown for HIV, tuberculosis, and malaria.^32^, ^33^

However, given shifts in the global development assistance landscape, the establishment of a new Global Fund-style financing mechanism for obesity and other non-communicable diseases appears unlikely under current geopolitical and economic conditions. This shift underscores the crucial importance of mobilising sustainable domestic financing and exploring innovative financing mechanisms, including public–private partnerships, health taxes, and results-based financing. Countries must harness these approaches, alongside continued international cooperation, to secure long-term, resilient funding for obesity prevention and care. As seen with other chronic conditions, placing people living with obesity at the centre of the response is crucial. Their involvement in advocacy, policy design, and service delivery can normalise care seeking, challenge stigma, and drive accountability. Lessons from HIV show that meaningful community involvement is not an adjunct but a core enabler of sustainable, rights-based health responses.

### Limitations

Although a key strength of the initiative was the introduction of a structured framework to guide policy development, support implementation, and enable scale-up, several limitations should be noted. Differences in national priorities, workforce capacity, and resource availability meant that countries engaged in the phases with varying levels of intensity, introducing variability in implementation fidelity. Reliance on self-reported progress against KPIs might also have introduced reporting bias, despite efforts to ensure consistency. The short timeframe of the 100-day challenge was valuable for catalysing action and accelerating the introduction of obesity prevention, care, and treatment services, establishing a foundation for scale-up. However, bringing chronic care programmes to scale requires sustained political commitment, long-term investment, and coordinated system-level approaches, which extend well beyond the challenge period. This requires continuing implementation follow-up, course correction, monitoring, and reprogramming. For this, outcome data, such as changes in obesity prevalence or service impact, are not yet available due to the early phase of implementation.

## Conclusions

The early results of the acceleration plan show that rapid progress is achievable when political will, structured implementation, and targeted technical support converge. Political commitment, robust stakeholder engagement, meaningful community participation, prolonged, targeted and innovative technical support, data-driven delivery, and long-term planning emerge as the core enablers of success. The 12 front-runner countries that completed phase 3 of the health system thematic deep dive offer provide a compelling blueprint for embedding obesity care within health systems at scale.

The next step is to transform this momentum into a global policy response that recognises obesity prevention, care, and treatment as both cost-effective public health imperatives and fundamental human rights. As seen with HIV, hepatitis C, immunisation, and more recently, diabetes and hypertension, the time has come for a coordinated global effort, led by national governments, to mobilise innovative and sustainable financing mechanisms and prepare systems that guarantee equitable access to obesity prevention and treatment in all countries. Without urgent action, the escalating burden of obesity and its comorbidities will continue to erode population health, widen health inequities, and undermine social and economic development worldwide.

### Contributors

## Declaration of interests

AW is Cabinet Member for Health, Wellbeing and Adult Social Care for the London Borough of Camden. All other authors declare no competing interests.
